# Predominant Dissemination of PVL-Negative CC89 MRSA with SCC*mec* Type II in Children with Impetigo in Japan

**DOI:** 10.1155/2011/143872

**Published:** 2011-12-07

**Authors:** H. Kikuta, M. Shibata, S. Nakata, T. Yamanaka, H. Sakata, K. Akizawa, K. Kobayashi

**Affiliations:** ^1^Pediatric Clinic, Touei Hospital, N-41, E-16, Higashi-ku, Sapporo 007-0841, Japan; ^2^Department of Pediatrics, Health Sciences University of Hokkaido, Kita-ku, Sapporo 002-8072, Japan; ^3^Nakata Pediatric Clinic, Shiroishi-ku, Sapporo 003-0023, Japan; ^4^Yamanaka Tatsuru Pediatrics, Shiroishi-ku, Sapporo 003-0022, Japan; ^5^Department of Pediatrics, Asahikawa Kosei Hospital, Asahikawa 078-8211, Japan; ^6^Department of Laboratory Medicine, Hokkaido University Medical Hospital, Kita-ku, Sapporo 060-0814, Japan; ^7^Department of Pediatrics, Sapporo Hokuyu Hospital, Shiroishi-ku, Sapporo 003-0006, Japan

## Abstract

*Background*. The ratio of CA-MRSA in children with impetigo has been increasing in Japan. *Methods*. Antimicrobial susceptibilities of 136 *S. aureus* isolates from children with impetigo were studied. Furthermore, molecular epidemiological analysis and virulence gene analysis were performed. *Results*. Of the 136 *S. aureus* isolates, 122 (89.7%) were MSSA and 14 (10.3%) were MRSA. Of the 14 MRSA strains, 11 belonged to CC89 (ST89, ST91, and ST2117) and carried diverse types of SCC*mec*: type II (IIb: 3 strains; unknown subtype: 4 strains), type IVa (2 strains), and unknown type (2 strains). The remaining three strains exhibited CC8 (ST-8)-SCC*mec* type VIa, CC121 (ST121)-SCC*mec* type V, and CC5 (ST5)-nontypeable SCC*mec* element, respectively. None were *lukS*-PV-*lukF*-PV gene positive. Gentamicin- and clarithromycin-resistant strains were frequently found in both MRSA and MSSA. *Conclusions*. PVL-negative CC89-SCC*mec* type II strains are the most predominant strains among the CA-MRSA strains circulating in the community in Japan.

## 1. Introduction

*Staphylococcus aureus* (*S. aureus*) is an etiologic agent for a wide range of illnesses from localized skin infections such as impetigo, furuncles, and cellulitis to life-threatening diseases such as pneumonia, meningitis, osteomyelitis, necrotizing fasciitis, endocarditis, toxic shock syndrome, and bacteremia [1]. Hospital-associated methicillin-resistant *S. aureus *(HA-MRSA) first appeared in 1961 shortly after the introduction of methicillin under the selective pressure of antibiotics [2, 3]. Since then, numerous MRSA clones have emerged and become a major cause of nosocomial infections throughout the world [4–6]. MRSA produces penicillin-binding protein 2′ (PBP2′) with a low affinity for *β*-lactam antibiotics. PBP2′ is encoded by the *mecA* gene, which is located on a large mobile genetic DNA element, the staphylococcal cassette chromosome *mec *(SCC*mec*) [7, 8]. Early MRSA clones were HA-MRSA isolated from patients with compromised immune systems under medical procedures. However, in 1993, community-associated MRSA (CA-MRSA) emerged in patients without risk factors in Western Australia [9]. CA-MRSA clones, which are distinct from HA-MRSA clones, have evolved differently in separate geographical areas and have spread in communities [10]. The incidence of infections due to CA-MRSA, including infections in children with no identifiable risk factors, has increased worldwide as a community pathogen [11, 12]. There are several types of CA-MRSA clones including ST1 (USA400) in Asia, Europe, and the US, ST8 (USA 300) in Europe and the US, ST30 in Australia, Europe, and South America, ST59 in Asia and the US, and ST80 (European clone) in Asia, Europe, and the Middle East. However, recent studies have shown that CA-MRSA strains are spreading into hospitals and are replacing traditional HA-MRSA strains [13, 14]. Therefore, distinction between the traditional HA-MRSA and CA-MRSA strains based on epidemiologic definitions has become difficult, and distinction based on the molecular typing is unclear [4, 15]. 

*S. aureus* is both a commensal organism and a pathogen. *S. aureus* colonized in the anterior nares, skin, and gastrointestinal tract plays an important role in transmission among individuals and in development of infection [16]. Hisata et al. and Ozaki et al. reported that the prevalences of nasal carriage of *S. aureus* in healthy Japanese children were 28.2% and 40.4%, respectively, and that the ratios of MRSA strains among the *S. aureus *isolates were 15.1% and 9.1%, respectively [17, 18]. Impetigo is a highly contagious infection of the superficial epidermis and is commonly caused by exfoliative toxins (ETs) of *S. aureus* [19]. The ratio of MRSA among *S. aureus* isolates from patients with impetigo between 1994 and 2000 was below 20% in Japan [20]. However, the ratio of MRSA has recently increased to 20 ~ 50% in Japan [21–24].

The characteristics of CA-MRSA strains in Japan are different from those reported in other countries. Although highly virulent CA-MRSA strains carrying Panton-Valentine leukocidin (PVL) genes have been spreading widely in the world, PVL-negative CA-MRSA has been disseminated in Japan [15, 17, 18, 23, 25]. The aim of this study was to investigate the molecular epidemiology by multilocus sequence typing (MLST) and staphylococcal cassette chromosome *mec *(SCC*mec*) typing and the antimicrobial susceptibilities of *S. aureus* isolates from children with impetigo. 

## 2. Materials and Methods

### 2.1. Bacterial Strains

Samples were isolated from skin swabs taken from pediatric outpatients with impetigo. A total of 136 *S. aureus* isolates were collected from 136 different patients (69 males and 67 females; mean age: 3.8 years; SD: 2.3 years) with impetigo at private offices of 25 practicing pediatricians and 10 hospitals between June 2009 and June 2010 in Hokkaido, the northernmost island of Japan. All samples were collected after obtaining informed consent from the children's parents. MRSA strains were identified by polymerase chain reaction (PCR) for the *mecA* gene and by the detection of penicillin-binding protein 2′ (Denka Seiken, Co., Tokyo, Japan).

### 2.2. Molecular Typing and Virulence Gene Analysis

PCR primers used for molecular typing and virulence gene analysis in this study are listed in Table 1. The presence of genes encoding PVL (*luk*S-PV-*luk*F-PV), toxic shock syndrome toxin 1 (TSST-1) (*tst*), and 2 ETs (ETA, ETB) (*eta*,* etb*) was examined by the PCR method using previously reported primers [26]. MRSA isolates were characterized by MLST as specific sequence types (STs) and clonal complexes (CCs) based on the DNA sequences at 7 defined loci. MLST was performed as described by Enright et al. [27]. MLST is based on sequence analysis of PCR products from seven *S. aureus *housekeeping genes, that is, *arcC, aroE, glpF, gmk, pta, tpi, and yqiL*. Each sequence was submitted to the MLST database website (http://www.mlst.net/) for the assignment of the allelic profile and ST. Different sequences are assigned distinct alleles of each housekeeping gene, and each isolate is defined by alleles of the seven genes. The ST data were further analyzed using eBURST (http://eburst.mlst.net/) to determine the CC. SCC*mec* typing was performed by the multiplex PCR method described by Kondo et al. SCC*mec* types (I to VI) were determined on the basis of the class (classes A, B, and C) of mec gene complex and the type (types 1, 2, 3, 4, and 5) of *ccr* gene complex [26]. Subtyping of SCC*mec* types II and IV was performed on the basis of different J1 regions by PCR using published primers [28].

### 2.3. Antimicrobial Susceptibility Testing

For *in vitro* antimicrobial susceptibility testing, minimal inhibitory concentrations (MICs) of a panel of 12 antimicrobial drugs, that is, oxacillin (MPIPC), ampicillin (ABPC), cefaclor (CCL), cefditoren (CDTR), clarithromycin (CAM), clindamycin (CLDM), fosfomycin (FOM), minocycline (MINO), gentamicin (GM), vancomycin (VCM), nadifloxacin (NDFX), and fucidic acid (FA), were determined using a frozen plate (Eiken Chemical Co., Tokyo, Japan). This plate is based on the broth microdilution method recommended by the Japan Society for Chemotherapy (JSC) [29, 30]. The breakpoints for these antimicrobial agents were determined according to the interpretation criteria of Clinical and Laboratory Standards Institute (CLSI) [31, 32] and JSC [29, 30]. Susceptibilities to antimicrobial drugs for which breakpoints are not determined by CLSI and JSC were defined in this study. A nitrocefin method was used for *β*-lactamase detection [33].

## 3. Results

In the bacteriological analysis of the 155 patients with impetigo, *S. aureus* and *Streptococcus pyogenes* were isolated from 136 and 6, respectively. Two cases involved two pathogens. No pathogenic organism was isolated from 15 patients. Of the 136 *S. aureus *cases, 122 (89.7%) were MSSA and 14 (10.3%) were MRSA. The 14 MRSA patients except for two newborns did not have a history of hospitalization during the previous year. Age distribution in the 136 patients was shown in Table 2.

The molecular characteristics of the 14 MRSA strains isolated from the patients with impetigo were examined and are summarized in Table 3. Among the 14 strains that carried the mec gene complex, 11 were determined the SCCmec element type. By SCC*mec* typing, the 14 MRSA strains were defined as SCC*mec* type II (IIb: 3 strains; II_NT_: 4 strains), type IVa (3 strains), type V (1 strain), and unknown type (3 strains). Types of *ccr* gene complex in 3 strains could not be determined. 

The 14 MRSA strains were comprised of 4 CCs. The four CCs were CC89 (11/14), CC5 (1/14), CC8 (1/14), and CC121 (1/14). ST89, ST91, and ST2117 strains belonged to the same CC89. No PVL-positive strains were identified in the 14 MRSA strains. The MRSA strains belonging to ST5 and ST8 carried TSST-1. Of the 14 MRSA strains, 11 (78.6%) were *etb *gene positive, and one was *eta* gene positive. There was no strain carrying both *eta* and *etb* genes. The MRSA strains belonging to ST5 and ST8 were both *eta* gene- and *etb* gene-negative.

Antimicrobial susceptibilities of the MRSA and MSSA strains to 12 antimicrobial agents are summarized in Table 4. The MICs of 12 antimicrobial agents for 14 individual MRSA strains are shown in Table 5. *β*-Lactamase was produced by 99 (72.8%) of the 136 *S. aureus* isolates. Of the 122 MSSA isolates, 85 (69.7%) were *β*-lactamase producers. All MRSA isolates were *β*-lactamase-positive. MRSA strains were highly resistant to CCL, CDTR, and CLDM. GM- and CAM-resistant strains were frequently found in both MRSA and MSSA. MINO, NDFX, FA and VCM had significant antimicrobial activity against the *S. aureus* isolates. The ST5 MRSA strain exhibited resistance to multiple drugs, including MINO and NDFX.

## 4. Discussion

The rate of CA-MRSA in patients with impetigo has been increasing in Japan. In this study, only 10.3% of the *S. aureus* isolates from patients with impetigo in Hokkaido, the northernmost island of Japan, were MRSA. The ratio of MRSA strains in Hokkaido is lower than those in other regions in recent studies [21–24]. This ratio of CA-MRSA in patients with impetigo in Hokkaido is similar to that in healthy children previously reported in Japan [17, 18]. However, further continuous surveillance is needed to clarify whether the low ratio of CA-MRSA in Hokkaido is true or due to biases.

HA-MRSA strains usually carry large SCC*mec* type I, II, or III and are usually multidrug resistant. On the other hand, CA-MRSA strains usually carry smaller SCC*mec* type IV or V and are usually more susceptible to non-*β*-lactam antibiotics [11, 34]. In previous studies, SCC*mec *type IV MRSA strains have been predominantly isolated from patients with impetigo [22, 35, 36]. Noguchi et al. reported that 98.7% of the MRSA strains isolated from patients with impetigo and staphylococcal scalded skin syndrome between 1999 and 2004 in Japan had type IV SCC*mec* [35]. Takizawa et al. reported that all of the characterized MRSA strains isolated from patients with impetigo in 2003 and 2004 also had type IV SCC*mec* [22]. Nakaminami et al. examined MRSA strains isolated from patients with impetigo in Japan in 2006 and reported that all isolates with SCC*mec *were classified into type IV (92.8%) and V (7.2%) [36]. MRSA strains isolated from children with impetigo in China from 2003 to 2007 were classified into type IV (54.5%), type V (18.2%), and VI (9.1%) [37].

However, in very recent studies, MRSA strains isolated from patients with impetigo had diverse SCC*mec* types, and SCC*mec* type IIb has been increasing [23, 24]. Type IIb SCC*mec *was first identified from MRSA strains isolated from healthy Japanese children [17]. Hisata et al. characterized 17 MRSA strains isolated from patients with impetigo at a Japanese hospital from June through August 2002. They found that the 17 MRSA strains carried diverse types of SCC*mec*: type II (IIb: 4 strains; unknown subtype: 2 strains), type IV (7 strains), and type V (4 strains) [23]. Shi et al. reported that among 26 MRSA strains isolated from patients with impetigo, 57.7% carried type IIa SCC*mec* element and 42.3% carried type IV SCC*mec *element [24]. In the present study, the MRSA strains isolated from patients with impetigo also had diverse SCC*mec* types, and SCC*mec* type II, including type IIb and II_NT_, was the predominant type among the CA-MRSA strains.

The role of PVL in the pathogenesis of staphylococcal infections is controversial [38]. However, PVL is responsible at least in part for the increased virulence of CA-MRSA. PVL-positive *S. aureus* strains are more frequently associated with cellulitis and abscesses than with impetigo [39–41]. In this study, none of the MRSA isolates carried the PVL gene. Although highly virulent CA-MRSA strains carrying PVL genes are known to prevail in the world, the prevalence of PVL-positive strains in Japan is not high [15, 17, 18, 23, 25]. There are three serological forms of ET, ETA, ETB, and ETD, which are linked to human impetigo [19]. The *eta* gene, encoding ETA, is located on a chromosome, whereas the *etb* gene, encoding ETB, is present on a plasmid. The **eta** and **etb** genes are generally found in **S. aureus** isolated from patients with impetigo. The *etb* gene is predominantly found in MRSA strains with **mecA*. *In this context, our data support previous data [21, 23, 24, 35, 36]. 

MLST is an excellent tool for investigating clonal evolution of MRSA. Six STs (ST5, ST8, ST89, ST91, ST121, and ST2117) were identified, and they were classified into four CCs (CC5, CC8, CC89, and CC121) in the present study. PVL-negative CA-MRSA strains, which belong to ST89 and ST91, have been associated with impetigo in Japanese children [22–24]. In this study, the most common ST in MRSA strains was also ST89 (57.1%), followed by ST91 (14.3%). ST89 and ST91 both belonged to the same CC89. We found that 11 of the 14 MRSA strains belonged to CC89 and carried diverse SCC*mec *types, IIb, II_NT_, Iva, and NT. Although one strain belonging to ST5, which has been commonly identified in HA-MRSA with type II SCC*mec*, was found in our MRSA isolates, it carried a nontypeable SCC*mec* element. The class of *mec* gene complex in the strain was class A, indicating that its SCC**mec** type should be a type other than type I, IV, V, VI, or VII [42]. In a previous study by Hisata et al. ST5 MRSA with SCC*mec* type IIa, which was indistinguishable from HA-MRSA strains (New York/Japan clone [5]) in Japan, accounted for 22.7% of MRSA strains isolated from healthy children [17]. Our ST5 MRSA strain was resistant to multiple drugs, including MINO and NDFX. Although ST8 MRSA strain with SCC*mec *type IVa [22, 43] and ST121 MRSA strain with SCC*mec *type V [44, 45] have been identified in CA-MRSA associated with impetigo, our MRSA strains with the same phenotypes did not carry PVL. 

It is generally accepted that bacterial resistance to antimicrobial agents parallels the frequency of use of the agents. Unlike HA-MRSA, CA-MRSA is generally susceptible to non-*β*-lactam antibiotics, such as aminoglycosides, tetracyclines, and fluoroquinolones. However, *S. aureus* isolates from patients with impetigo were highly resistant to GM and CAM, reflecting frequent usage of GM ointment for the treatment of skin infections including impetigo and frequent usage of CAM for respiratory infection in Japan [20, 21, 35, 36].

## 5. Conclusions

This study and a previous study indicated that PVL-negative CC89 strain with a marked divergence in SCC*mec* types predominated in Japanese communities, suggesting that it might be a Japanese domestic CA-MRSA clone. The existence of multiple types of SCC*mec* elements suggested that MRSA strains might be emerging locally in the community under the selective pressure of antibiotics. Furthermore, SCC*mec* II CA-MRSA may at least partially be beginning to replace SCC*mec* IVa CA-MRSA. Continuous monitoring of MRSA epidemiology using MLST and SCC*mec* typing and drug resistance trends would show change in the clonal distribution of MRSA, which may be crucial for the effective management of impetigo.

## Figures and Tables

**Table 1:** List of primers.

Gene	Primer name	Size of product (bp)	Primer sequence (5′→3′)	Reference
(1) Detection of virulence gene				[26]
*lukS-PV-lukF-PV*	luk-PV-1		ATCATTAGGTAAAATGTCTGGACATGATCCA	
	luk-PV-2	433	GCATCAASTGTATTGGATAGCAAAAGC	
*tst*	TST-1		TTCACTATTTGTAAAAGTGTCAGACCCACT	
	TST-2	180	TACTAATGAATTTTTTTATCGTAAGCCCTT	
*eta*	mpETA-1		ACTGTAGGAGCTAGTGCATTTGT	
	mpETA-3	190	TGGATACTTTTGTCTATCTTTTTCATCAAC	
*etb*	mpETB-1		CAGATAAAGAGCTTTATACACACATTAC	
	mpETB-2	621	AGTGAACTTATCTTTCTATTGAAAAACACTC	
(2) SCC*mec* typing				[28]
(i) *ccr* gene complex type with* mecA *				
*mecA*	mA1		TGCTATCCACCCTCAAACAGG	
	mA2	286	AACGTTGTAACCACCCCAAGA	
Type 1	*α*1		AACCTATATCATCAATCAGTACGT	
	*β*c	695	ATTGCCTTGATAATAGCCITCT	
Type 2	*α*2		TAAAGGCATCAATGCACAAACACT	
	*β*c	937	ATTGCCTTGATAATAGCCITCT	
Type 3	*α*3		AGCTCAAAAGCAAGCAATAGAAT	
	*β*c	1,791	ATTGCCTTGATAATAGCCITCT	
Type 4	*α*4.2		GTATCAATGCACCAGAACTT	
	*β*4.2	1,287	TTGCGACTCTCTTGGCGTTT	
type 5	*γ*F		CGTCTATTACAAGATGTTAAGGATAAT	
	*γ*R	518	CCTTTATAGACTGGATTATTCAAAATAT	
(ii) *mec* gene complex class				
*mecA*	ml6		CATAACTTCCCATTCTGCAGATG	
	mA7	1,963	ATATACCAAACCCGACAACTACA	
*mecB*	IS7		ATGCTTAATGATAGCATCCGAATG	
	mA7	2,827	ATATACCAAACCCGACAACTACA	
*mecC (C2)*	mA7		ATATACCAAACCCGACAACTACA	
	IS2(iS-2)	804	TGAGGTTATTCAGATATTTCGATGT	
(iii) SCC*mec* type II subtyping				
IIa (II.1)	kdpB2		TAAACTGTGTCACACGATCCAT	
	kdpB1	287	GATTACTTCAGAACCAGGTCAT	
IIb (II.2)	2b3		GCTCTAAAAGTTGGATATGCG	
	2b4	1,518	TGGATTGAATCGACTAGAATCG	
IIE (II.3)	4b3		AACCAACAGTGGTTACAGCTT	
	4b4	726	CGGATTTTAGACTCATCACCAT	
II.4	II4-3		GTACCGCTGAATATTGATAGTGAT	
	II4-1	2,003	ACTCTAATCCTAATCACCGAAC	
(iv) SCC*mec* type IV subtyping				
IVa (IV.1)	4al		TTTGAATGCCCTCCATGAATAAAAT	
	4a3	458	AGAAAAGATAGAAGTTCGAAAGA	
IVb (IV.2)	4b3		AACCAACAGTGGTTACAGCTT	
	4b4	726	CGGATTTTAGACTCATCACCAT	
IVc (IV.3)	4c5		ATCCATTTCTCAGGAGTTAG	
	4c4	259	AGGAAATCGATGTCATTATAA	
IVd (IV.4)	4d3		AATTCACCCGTACCTGAGAA	
	4d4	1,242	AGAATGTGGTTATAAGATAGCTA	
(3) Multilocus sequence typing (MLST)				[27]
carbamate kinase (*arcC*)	arcC-Up		TTGATTCACCAGCGCGTATTGTC	
	arcC-Dn	456	AGGTATCTGCTTCAATCAGCG	
Shikimate dehydrogenase (*aroE*)	aroE-Up		ATCGGAAATCCTATTTCACATTC	
	aroE-Dn	456	GGTGTTGTATTAATAACGATATC	
Glycerol kinase (*glpF*)	glpF-Up		CTAGGAACTGCAATCTTAATCC	
	glpF-Dn	465	TGGTAAAATCGCATGTCCAATTC	
Guanylate kinase (*gmk*)	gmk-Up		ATCGTTTTATCGGGACCATC	
	gmk-Dn	429	TCATTAACTACAACGTAATCGTA	
Phosphate acetyltransferase *(pta*)	pta-Up		GTTAAAATCGTATTACCTGAAGG	
	pta-Dn	474	GACCCTTTTGTTGAAAAGCTTAA	
Triosephosphate isomerase (*tpi*)	tpi-Up		TCGTTCATTCTGAACGTCGTGAA	
	tpi-Dn	402	TTTGCACCTTCTAACAATTGTAC	
Acetyl coenzyme A acetyltransferase *(yqiL*)	yqiL-Up		CAGCATACAGGACACCTATTGGC	
	yqiL-Dn	516	CGTTGAGGAATCGATACTGGAAC	

**Table 2:** Age distribution in the 136 patients.

Age (years)	MSSA	MRSA	Total
*＜*1	13	2	15
1–<2	19	2	21
2–<3	16	5	21
3–<4	16	2	18
4–<5	15	1	16
5–<6	17	0	17
6–<7	15	2	17
7–<12	11	0	11

Total	122	14	136

**Table 3:** Molecular characterization of 14 MRSA strains isolated from patients with impetigo.

Strain	Age	SCC*mec* typing			MLST			Presence of exotoxin genes			
		SCCmec type	*ccr*	*mec*	ST	CC	Allelic profile	*lukS-PV-lukF-PV*	*tst*	*eta*	*etb *
T-11	1 yr	IIb	2	A	2117	89	1-310-28-18-18-33-50	−	−	−	+
T-33	6 yrs 4 mos	IVa	2	B	91	89	1-26-28-18-18-54-50	−	−	−	+
T-55	2 yrs 6 mos	NT	NT	A	89	89	1-26-28-18-18-33-50	−	−	−	+
T-64	2 yra 6 mos	II_NT_	2	A	89	89	1-26-28-18-18-33-50	−	−	−	+
T-71	6 yrs 6 mos	IIb	2	A	89	89	1-26-28-18-18-33-50	−	−	−	+
T-79	3 yrs 10 mos	IVa	2	B	91	89	1-26-28-18-18-54-50	−	−	−	+
T-83	3 yrs 11 mos	II_NT_	2	A	89	89	1-26-28-18-18-33-50	−	−	−	+
T-114	2 yrs 3 mos	NT	NT	A	89	89	1-26-28-18-18-33-50	−	−	−	+
T-119	2 yrs 2 mos	NT	NT	A	5	5	1-4-1-4-12-1-10	−	+	−	−
T-125	4 yrs	V	5	C2	121	121	6-5-6-2-7-14-5	−	−	+	−
T-132	19 ds	II_NT_	2	A	89	89	1-26-28-18-18-33-50	−	−	−	+
T-137	1 yr 1 mo	IIb	2	A	89	89	1-26-28-18-18-33-50	−	−	−	+
T-139	10 ds	II_NT_	2	A	89	89	1-26-28-18-18-33-50	−	−	−	+
T-144	2 yrs 1 mo	IVa	2	B	8	8	3-3-1-1-4-4-3	−	+	−	−

NT: nontypeable.

**Table 4:** Comparison of antimicrobial susceptibilities of MRSA and MSSA.

Antimicrobial agent	Resistance breakpoint (*μ*g/mL)	MSSA (*n* = 122)			MRSA (*n* = 14)		
		Range (*μ*g/mL)	MIC_90_ (*μ*g/mL)	Resistance (%)	Range (*μ*g/mL)	MIC_90_ (*μ*g/mL)	Resistance (%)
MPIPC	4	≦2	≦2	0	4–>8	>8	100
ABPC	0.5	≦0.12 5–>16	16	71.3	4–>16	>16	100
CCL	16	1–16	4	1.6	4–>16	>16	92.9
CDTR	2*	0.5 –2	1	0.8	2–>16	>16	100
CAM	4	≦0.125–>16	>16	67.2	>16	>16	100
CLDM	1	≦0.125–0.25	0.25	0	≦0.125–>16	>16	71.4
FOM	8**	0.25–>16	16	20.5	1–>16	8	14.3
MINO	8	≦0.125–0.25	≦0.125	0	≦0.125–16	0.25	7.1
GM	8	0.25–>16	>16	54.1	1–>16	>16	92.9
VCM	4	≦0.5–1	1	0	1-2	1	0
NDFX	4**	≦0.125–2	≦0.125	0	≦0.125–>16	≦0.125	7.1
FA	2**	≦0.125–>16	0.5	0.82	≦0.125–1	0.5	0

The breakpoints for these antimicrobial agents were determined according to the interpretation criteria of Clinical and Laboratory Standards Institute (CLSI). Susceptibility to antimicrobial drugs for which breakpoints are not determined by CLSI was according to the interpretation criteria of the Japan Society for Chemotherapy (*) or defined in this study (**).

**Table 5:** Antimicrobial susceptibilities of 14 MRSA strains.

Strain	MIC (*μ*g/mL) of antimicrobial agents											
	MPIPC	ABPC	CCL	CDTR	CAM	CLDM	FOM	MINO	GM	VCM	NDFX	FA
T-11	>8	>16	>16	>16	>16	>16	1	≦0.125	>16	2	≦0.125	0.25
T-33	>8	16	>16	4	>16	>16	2	≦0.125	>16	1	≦0.125	0.25
T-55	>8	16	>16	8	>16	>16	4	0.25	>16	1	≦0.125	0.25
T-64	>8	>16	>16	>16	>16	>16	2	≦0.125	>16	1	≦0.125	0.25
T-71	>8	16	16	4	>16	>16	2	≦0.125	>16	1	≦0.125	≦0.125
T-79	>8	16	>16	4	>16	≦0.125	4	≦0.125	>16	1	≦0.125	0.25
T-83	>8	16	>16	4	>16	>16	2	0.25	>16	1	≦0.125	≦0.125
T-114	>8	16	>16	16	>16	>16	1	≦0.125	>16	1	≦0.125	0.25
T-119	>8	>16	>16	>16	>16	≦0.125	>16	16	>16	1	>16	0.5
T-125	4	4	4	2	>16	0.25	4	≦0.125	>16	1	≦0.125	0.25
T-132	>8	16	>16	16	>16	>16	2	≦0.125	>16	1	≦0.125	≦0.125
T-137	>8	>16	>16	>16	>16	>16	2	≦0.125	>16	1	≦0.125	1
T-139	>8	16	>16	16	>16	>16	2	≦0.125	>16	1	≦0.125	0.25
T-144	>8	16	>16	16	>16	≦0.125	8	≦0.125	1	1	≦0.125	0.25
